# Guidelines for the Revision and Use of Revised Psychological Tests: A Systematic Review Study

**DOI:** 10.5964/ejop.2901

**Published:** 2022-08-31

**Authors:** Johan H. Cronje, Mark B. Watson, Louise-Anne Stroud

**Affiliations:** 1 Department of Psychology, Nelson Mandela University, Gqeberha, South Africa; University of Wrocław, Wrocław, Poland

**Keywords:** test revision, guidelines, psychological tests, revised tests

## Abstract

Tests are updated and revised periodically in order to remain current, valid and reliable in a competitive psychological testing industry. Despite the prevalence of test revisions, especially in recent years, a number of authors have commented on the lack of comprehensive guidelines for test revision. Whilst some guideline documents from test associations have mentioned test revision, these guidelines tend to be focussed on test user responsibility, with limited guidance for practitioners embarking on a test revision project. Test revision is expensive and time consuming, leaving little scope for experimentation or trial-and-error. Test revision deserves a comprehensive document that addresses aspects such as what the different types of revision are, when to embark on a revision, what process to follow and how test users should use revised tests. The current study developed a comprehensive and practical set of 23 guidelines across ten phases of a revision project to assist revision teams, test users and publishers. These guidelines were peer-reviewed and refined.

Revised editions of psychological tests have appeared regularly in the past two decades. Despite this, national and international test organisations have not provided clear guidelines for revision teams or test users that cover the lifespan of revision projects. A search for guidelines from organisations, such as the International Test Commission (ITC), American Psychological Association (APA), American Educational Research Association (AERA), European Federation of Psychologists’ Associations (EFPA), and Education Testing Service (ETS), indicates that these organisations have focussed on guidelines related to fair and ethical test use (AERA, 2014; ETS, 2009, 2014; International Test Commission, 2013a). The ITC has been the most prolific organisation in generating guidelines regarding technical aspects of psychological testing such as test translation and adaptation (2017), test security (2014), and computer-based and internet-delivered tests (2005). The most comprehensive guidelines for test revision are within the *Guidelines on Practitioner Use of Test Revisions, Obsolete Tests, and Test Disposal* published by the International Test Commission (2015). These, in brief, address three areas, namely the relationship between test publisher and test users, the communications from test publishers to test users, and the responsibilities of test users in relation to revised tests.

These guidelines do not address the process of test revision thereby implying that those involved in such projects should be well versed in the practice as there are similarities between test construction, adaptation, and revision. Thus, practitioners involved in test revision can find some direction by reading different test guidelines. However, test revision presents unique challenges that practitioners engaged in such projects should be aware of. These include the expectations of existing test users, the increased economic pressures placed on the revised test by its owner or publisher, and the difficulty of the revised test’s relationship to its possibly well-regarded predecessor.

More experienced practitioners may navigate their way successfully through a test revision by using guideline statements from different documents. Many aspects are not directly addressed, however, by existing guidelines, making it difficult for novice practitioners to navigate a test revision process. Utilising different guideline documents is also problematic, as the intentions of some statements may contradict each other, and practitioners could decide to adhere to only some guidelines. A 2000 issue of the APA journal, *Psychological Assessment,* called for comprehensive guidelines on test revision (Adams, 2000; Butcher, 2000; Silverstein & Nelson, 2000; Strauss, Spreen, & Hunter, 2000), a call that nearly two decades later has not been addressed by test organisations.

Test revision guidelines could be comprehensive and cover aspects such as what the different types of revision are, when to embark on a revision, what process to follow and how test users should use revised tests.

The reality of developing such comprehensive guidelines is the challenge of balancing aspects that may be self-evident and important for test revision, with the need for robust guidelines focussed on actions that can be controlled by revision teams, and for which they can be held accountable. Examples of important, yet difficult to control suggestions would be that:

- Revision teams should consist of a mix of internal and external stakeholders of the test (ETS, 2009; Foxcroft, 2004).
- Agreements about ownership, revision budget and royalties should be decided early in the revision process (Butcher, 2000).
- The economic circumstances of test users should be considered when determining the cost of a revised test (Adams, 2000; Camara, 2007; Naglieri et al., 2004).
- Test publishers and users share a joint responsibility to engage with each other regarding revised tests (Adams, 2000; International Test Commission, 2015).
- Test publishers should develop a reasonable strategy to assist test users to switch to a revised test edition (Bush, 2010; International Test Commission, 2015).
- Test publishers should offer comprehensive training to promote the level of competence with which test users employ revised tests (Butcher, 2000; EFPA, 2013b; International Test Commission, 2013a).
- Test users should guard against resistance to change, keep current with changes to tests, and strive to adopt a revised test as soon as possible, with due consideration for the best interests of their clients (Bush, 2010; Butcher, 2000; International Test Commission, 2015; King, 2006).

The above guidelines are worthy of notice, but dependent on the specific test being revised, the scope of the revision, the level of influence of the revision team on financial matters and ownership, and the specifics surrounding the decisions made by test users.

Guidelines differ from standards or policies. Guidelines are most likely created by experts to provide practical guidance for practitioners, that they can opt to adhere to or not. Rosen, Proctor, and Staudt (2003) define guidelines as “systematically compiled and organised knowledge statements to help practitioners select and use the most effective and appropriate interventions for attaining desired outcomes” (p. 209). Standards are adopted by organisations, thereby creating a level of compliance from members. According to the APA (2017), standards tend to focus on broader issues such as acting with competence, dealing with ethical dilemmas, exercising respect for others, maintaining confidentiality, the right to privacy, seeking informed consent, and maintaining adequate records. Policies are more direct and enforced by organisations. A *Certified Information Systems Auditor* study (CISA, 2011) offers insight into these terms from an institutional perspective. According to CISA (2011), policies are considered high-level documents that exercise control over staff, and they are usually enforced at managerial level. Given the scarcity of standards or policies for test revision, the researchers embarked on the present study to provide such guidance on the process of test revision.

## Method

The systematic review was performed according to the standards set by Moher, Liberati, Tetzlaff, Altman, and The PRISMA Group (2009). A systematic review from 2000–2017 was conducted of existing standards and guidelines published by authors and organisations. Documents were sourced using relevant keyword searches [psycho* AND test* OR measure* AND revis* AND guide* OR standard* OR polic*] in online databases (including EBSCOhost, Findplus, Sabinet, Science Direct, Springer, Taylor & Francis, and Wiley Online Library). The search terms reflect that only resources in English were included in the study, which is a limitation of the research. The database search results and number of resources that were included in the review are displayed in Table 1.

**Table 1:** Database Search Results

Database	Resources Found	Included in Systematic Review
EBSCOhost	242	4
FindPlus	325	1
Sabinet	94	1
Science Direct	275	1
Springer	577	0
Taylor & Francis	248	0
Wiley Online Library	64	0
**Total**	**1825**	**7**

To increase the number of sources the researchers expanded the search to include websites of national and international test organisations, and conference proceedings. An additional filter was applied to remove duplicate documents, limit author bias and to conduct a quality check for institutional endorsement or peer-review. In all, 21 original resources were included in the systematic review, to highlight themes and extract guidelines. The authors used the results of the systematic review, together with their experience in test construction and revision to build on the relevant information contained within the 21 sources to develop 23 guidelines across ten phases of test revision as conceptualised by the researchers. The guidelines were submitted for peer review to an international panel of seven practitioners with experience in test construction or revision. Feedback from the panel was used to refine the guidelines. Each guideline starts with a broad topic statement that is explained in the subsequent text. The guidelines are discussed according to the phases of test revision they relate to, but as they represent overarching themes there may be some overlap or repetition of key messages throughout the explanatory texts.

## Guidelines

### Phase One: Pre-Planning

**1.1** *Test revisions should endeavour to improve the quality, utility, accuracy, reliability and fairness of a test.* After obtaining permission from the test’s owner to conduct a test revision, a revision team should take cognisance of preceding versions of the test and the body of research evidence and test user feedback. Different aspects that can be revised in a test include: refinements to the underpinning construct of the test, the relevance of stimuli, and normative information, an extension of the age range of the test population, broadening the intended test population in terms of ethnic, cultural or language groups, improved accuracy and reliability of the test, and alternative forms of administration, scoring and reporting (Bush 2010; Strauss, Spreen, & Hunter, 2000).

### Phase Two: Initial Investigation

**2.1** *Test publishers are responsible for monitoring the context within which tests operate, including the use of and feedback about tests, and the industry requirements for psychological tests, as this information may inform the decisions of revision teams.* Test publishers have a responsibility to monitor changes in test conditions and the use of their test products (AERA, 2014). Changes to industry standards may require a publisher to revise a test to align it with the updated standards. If significant test information or content has been published within the public domain, it may challenge test validity, which will require test revision earlier than anticipated (Naglieri et al., 2004). Publishers should be proactive in seeking feedback from test users and researchers (Adams, 2000). In the event that any changes to the use of a test are made, test users should be informed of the changes that affect them, including the intention to embark on a revision project (ETS, 2014).

**2.2** *A test should be revised or withdrawn when new research data, significant changes in the test domain, or altered conditions of test use may affect the validity of test score interpretations.* It can be challenging to choose the correct moment to revise a test. An important cue is when critical test components have become outdated (Adams, 2000). A key indicator that this has occurred is changes to the theoretical framework that underpins the test. In addition to this, advances in measurement theory, psychological testing practice, and norm development are also important considerations (King, 2006). Changes in the intended test population over time may also necessitate a change. Publishers should remain cognisant of changes to important industry standards and benchmark their products against them.

**2.3** *During a test revision, feedback should be obtained from diverse internal and external sources, including test users and test takers.* It is important to gather feedback from test users and researchers early in the project regarding changes that are required in the test (Butcher, 2000). Requesting input serves multiple functions. Firstly, it recognises and values the experience of test users and makes them feel included in the revision. Secondly, it allows for identification of latent experts on the test, who may be drawn on during later phases of the revision project (International Test Commission, 2013b). Thirdly, it creates a sense of collaboration between the revision committee and test users. Finally, it creates a database of interested users and researchers who may be approached later to review the revised test and to provide feedback on the likely acceptance of the product by the broader market (Adams, 2000; ETS, 2014).

### Phase Three: Project Planning

**3.1** *Revision teams should provide a plan to address fairness in the design, development, administration, and use of a revised test.* The ultimate goal of a psychological test is to measure a construct or set of constructs accurately and fairly, without any interference from sources that are not integrally linked to the construct(s). The intended changes of a test revision should include therefore plans to improve fairness and accuracy (ETS, 2009). For a current test revision, the measures taken to improve fairness, validity and reliability, including the analyses used and results thereof should be documented (AERA, 2014).

**3.2** *The rationale, goals, scope, and process of a test revision should be planned, followed and documented.* The goals and scope of the revision project should be delineated at the outset to act as a compass. Each step in the process should be documented to demonstrate how technical quality has been achieved (International Test Commission, 2013b). The rationale for major decisions about the current test revision should be explained in detail, as these will be important for existing users of previous versions of the test, as well as for future revisions of the test (ETS, 2014).

### Phase Four: Academic Enquiry

**4.1** *The conceptualisation and operationalisation of components of revised tests should be reviewed and appropriately revised to minimise construct-irrelevant sources of score variance.* The variance in test scores should be linked directly to variance in the assessed construct, and not because of construct-irrelevant sources (Camara, 2007). As such, performance should provide valid evidence of the test construct for test takers from all populations for whom the test was designed (International Test Commission, 2013a; Oliveri, Lawless, & Young, 2015). Revision teams should conduct research to determine the extent of construct-irrelevant interference in test scores, as such interference may affect the recommendations that are based on test scores (ETS, 2009, 2014). Culture and language are important considerations in this regard, as they can inform the choice of specific words or phrases, as well as item formats and modes of testing (Foxcroft, 2004).

**4.2** *Revision teams should balance the needs of test users and the domain measured, when deciding on test items and the nature of tasks required from test takers of a revised test.* As part of the academic enquiry of a test revision, revision teams should familiarise themselves with the needs of practitioners who use the test. It is important to understand the contexts in which a test is used, as well as for what purpose. The nature of tasks included in a revised test should be informed by the contexts in which the test is utilised, as well as by the test takers (Liu & Dorans, 2013).

**4.3** *Utilising careful analysis, optimally functioning components of a test should be considered for inclusion in a revised test to act as anchor items, or to foster a sense of brand familiarity between different test editions.* The product of a major revision that reflects a shift in underpinning constructs, test questions, target populations, as well as scoring or norming methods, can create a sense of disconnect between the revised and previous test versions. Steps to address this potential lack of connection are to include items from previous versions in a revised test to create an anchor block, which can assist in establishing the link in test difficulty between different versions (Geisinger, 2013), or to retain the item formats and scoring systems of the previous version, to minimise administrator error (Adams, 2000).

### Phase Five: Item Development

**5.1** *The development of test items should consider multicultural contexts, and the possibility that revised tests may be used eventually in settings for which they were not initially intended.* A popular test may be used eventually in contexts and countries for which it was not originally designed. Revision teams need to be aware of this possibility and develop items that either are applicable for a global audience or easily adapted for other cultures (Foxcroft, 2004, International Test Commission, 2013a). Another trend in psychological testing is the conversion of standard tests to computer-based or online tests. These modes of testing require special considerations and adaptations. The equivalence of traditional and technological versions of a revised test would be improved if revision teams were mindful of such future developments, and if they created test items from the outset that could be extended to other modes of testing (Strauss, Spreen, & Hunter 2000).

**5.2** *When authoring item content and test instructions, revision teams should anticipate translation of a revised test into other languages in the future.* A popular practice in the test industry is to translate tests into other languages to extend the test user market and for cross-cultural research. Multiple-language tests are not only desirable, but also often necessary to reduce bias and promote accurate and fair testing in international settings (Geisinger, 2013). Translation from the original source language to a new target language without accounting for cultural differences can be a significant source of construct-irrelevant interference. Test translations should be performed by qualified experts to minimise language bias as a nuisance variable. Revision teams should provide evidence of the similarity in meaning and difficulty levels of test questions for all intended populations for a revised test (International Test Commission, 2017; Oliveri, Lawless, & Young, 2015).

### Phase Six: Test Piloting

**6.1** *Test items and equipment must be field-tested and piloted sufficiently using samples that represent the intended population for the revised test.* In test revision, there is a chance that the final item mix in a test will consist of newly developed items, intact items from the previous version, and items from the previous version that have been updated or refined. Revision teams should not rely on assumptions as a basis for final item selection and placement in the revised test, but all decisions should be informed by field-testing and pilot studies (Butcher, 2000). The purpose of field-testing is to obtain feedback from test takers and users, which can be utilised to refine items. It also assists in quality control by detecting errors in the administration, content, and scoring of items (Camara, 2007). Piloting is used mainly to collect quantitative data on a pool of potential test items, to allow for item analysis and to assist in the selection of items for the final revised test (International Test Commission, 2017). It is advisable that samples for field-testing and piloting closely resemble the intended test population (AERA, 2014).

**6.2** *Revision teams should select a balanced mix of items for a revised test to ensure that all intended underpinning constructs are adequately assessed at various ability levels.* Selecting appropriate items for inclusion in a revised test is crucial. Consideration should be given to user needs, test length, and coverage of underlying constructs at all intended levels of difficulty. The number of test items will depend on the focus of the test, as screening tests may require fewer items per construct than diagnostic tests (Liu & Dorans, 2013). For revised tests that provide broader assessment of a construct, evidence should be provided to prove even coverage of the test construct and its ability to assess the knowledge, skills, and abilities of test takers (Oliveri, Lawless, & Young, 2015).

### Phase Seven: Test Standardisation

**7.1** *Revision teams should give due consideration to the representativeness and size of standardisation samples in order to develop normative information for a revised test that is applicable to intended test takers.* Revision teams should design a strategy to develop norms that maximise generalisability and usability, whilst keeping costs within acceptable parameters (Butcher, 2000). The norm sample should consist of participants that are relevant for the intended test populations. In the event that the norm sample cannot consist of sufficient representation from all groups, research should be conducted to demonstrate the equivalence in performance of different groups on a revised test (International Test Commission, 2013a, 2017). Revision teams should consider sample size from test classification agencies. All information about size, composition, and source of norm groups, including their representativeness, should be provided in test manuals (EFPA, 2013a).

**7.2** *Revised tests should be accompanied at launch with adequate norms and standardisation information.* Revised tests should be published with the relevant documentation and information that would allow test users to determine the suitability of a test for their clients. The standard information required includes evidence to support the norms, and the validity and reliability of the revised test for the intended populations (International Test Commission, 2017). Some tests are used to assist in the diagnosis of certain disorders or illnesses, and to monitor the effectiveness of treatment for clients. With the fragmentation of traditional diagnoses into ever-widening and deepening layers, producing norms or research relevant to each category may be unfeasible. Test manuals should therefore provide at least some information about the scores of test takers from certain clinical groups, compared with matched samples from non-clinical samples (Geisinger, 2013).

### Phase Eight: Conduct Supporting Research

**8.1** *Revision teams should prioritise research into all target populations of a revised test, including clinical and non-clinical samples*. It may take years after publication for research to be conducted with a revised test on clinical populations. Revision teams should identify key populations and conduct research for such populations, for inclusion in the test manuals and training materials. Research should draw on samples from various clinical and non-clinical populations, and effort should be made to produce research that will maximise the usability and generalisability of findings (Oliveri, Lawless, & Young, 2015). Users of revised tests should request research information on clinical populations from test publishers, and consider contributing to such projects (Bush, 2010).

**8.2** *Multiple methods should be employed to investigate the relationship between previous and revised editions of a test*. It is important for test users to understand how a revised test compares to its predecessors. Failure to do so would lead to misleading results, and result in unintended and inappropriate use of a revised test (Strauss, Spreen, & Hunter, 2000). This information includes a comparison of the validity and reliability of the previous and revised editions, differences in the intended populations, conditions for test use, administration and scoring guidelines, and how norm tables should be used and results interpreted.

**8.3** *Research should be conducted into the validity and reliability of a revised test*. Revision teams have a responsibility to provide comprehensive evidence of the test validity and reliability of a revised test (Butcher, 2000). This information should include technical documentation that highlights different types of validity and reliability (Camara, 2007). Research is ever expanding in these fields, but revision teams should focus on tried-and-tested methods that communicate the strengths and weaknesses of a revised test in a clear and unbiased fashion (Mattern, Kobrin, & Camara, 2012).

### Phase Nine: Test Product Assembly and Launch

**9.1** *The extent of a revision should be communicated in the product description of a test.* Butcher (2000) identifies ‘light’, ‘medium’ and ’extensive’ as three types of test revision. A ‘light’ revision entails changes made mostly to the test manual, such as minor updates to item wording or editorial changes. A ‘medium’ revision is more intensive and includes changes to or replacing non-performing items, and updating the norms of a test. An ‘extensive’ revision involves a complete reanalysis and reconstruction of the test. This could include re-examining the theoretical foundation of the test and major changes to items or subscales, together with a new set of test instructions. An extensive revision would also include new norm data, as well as validity and reliability studies (Butcher, 2000). The term ‘revised’ should only be attached to tests that have been updated in significant ways, such as in ‘medium’ and ‘extensive’ revisions. If the test has not been changed significantly after a ‘light’ revision, the test should rather be marketed as containing minor changes or updates (AERA, 2014).

**9.2** *When tests are revised, users should be informed of the changes to the specifications, underlying constructs, and changes to the scoring method.* Revision teams should present any changes to a revised test in comprehensive technical documentation including how the revised test differs from its predecessor (International Test Commission, 2017). The theoretical foundations for updates to constructs should be supplied (EFPA, 2013a). Any differences in target populations, methods of norm development, and the correspondence between norms from previous and revised test editions and their potential impact should be unpacked (International Test Commission, 2015). Emphasis should be placed on evidence regarding how the revised test builds or improves on its predecessor (Naglieri et al., 2004).

**9.3** *Test users should be clearly informed of the comparability and relationship between the previous and revised editions of a test.* There are many reasons why the ties between the previous and revised editions of a measure should be clearly established. The first is that a revision team may face change resistance from established test users (Butcher, 2000). The second reason is that test users conduct an assessment based on the construct in question and should be made aware of the comparability of the constructs between previous and revised test versions (EFPA, 2013b). A third motivation is that, despite following explicit blueprints in test revision, changes may occur over time, as it is more difficult to develop, clone or replicate some items for a revised test (International Test Commission, 2013b). This could affect the overall difficulty of the revised test, which will affect how its test scores compare to a previous version (Liu & Dorans, 2013).

**9.4** *Documentation for revised tests should be amended and dated to keep information for test users current.* Any substantial changes to a test should be reflected in its updated documentation, and with supplementary information to existing test users. This includes general information as well as cautions regarding test use (AERA, 2014). The focus should be on the adequacy of information for test users, including administration guidelines, technical information, and norm supplements (EFPA, 2013a).

### Phase Ten: Post-Launch Activities

**10.1** *Revision teams should develop a comprehensive post-launch research strategy and encourage the dissemination of independent research studies.* As a revised test is adopted by test users, it is used in many contexts with test takers from different backgrounds. Each test session is unique and provides an opportunity for research and learning. Revision teams should spearhead ongoing research into a revised test. They should develop a list for test users and researchers that highlights the evidence required to validate a revised test for use on different populations (Mattern, Kobrin, & Camara, 2012). In addition, revision teams should encourage independent research aimed at replicating the validity and reliability claimed in test materials (International Test Commission, 2013b). Test users should be open to participating in research studies (International Test Commission, 2013a).

## Discussion

These 23 guidelines provide guidance for stakeholders of test revision, including revision teams, test publishers, and test users. The guidelines highlight 8 pertinent themes on test revision:

1. The various reasons to revise a test, including factors that are internal to the test, and aspects that constitute the external environment that a test operates in.
2. The participation of different role players throughout a test revision.
3. Communication between publishers, revision teams, test users, and researchers.
4. The continuing role of planning to determine the scope and process during a test revision, and post-launch activities.
5. The relationship between previous and revised test editions.
6. Considering fairness towards different cultural and language groups when selecting test items.
7. Validity and reliability evidence that highlight the fairness of the test.
8. Maximising the generalisability of the test norms and interpretation of test results.

It is worth noting that the guidelines in the last two phases refer to the launch and continued responsibility of developers beyond the launch of the test. These extend beyond the extant standards and guidelines on test revision from notable organisations such as the AERA, APA, ETS, and International Test Commission, who provide few guidelines on these areas (AERA, 2014; ETS, 2009, 2014; International Test Commission, 2013a, 2013b, 2015, 2017). Whilst the guidelines in the last two phases referred to existing guidelines from these organisations, these organisations did not create a clear link in their guidelines to test revision. This means that practitioners engaged in test revision may not be aware of these guidelines. Further, the relative silence of these organisations on the responsibilities of revision teams and publishers after a test is launched may add to a misconception amongst test users and less experienced revision teams that a revision journey ends with the revised test’s launch. The present guidelines highlight however that a revision can be viewed as a precursor to the work that follows the launch. The success of a revised test depends on the effort that goes into the marketing, training and follow-up that occurs after it enters the test market (Geisinger, 2013). At the point of launch, a revised test enters the test user market. Some questions and issues will initially surface in practical daily test sessions between test users and test takers (Silverstein & Nelson, 2000). This will necessitate communication with test publishers and the refinement of some revised test components by revision teams. A final comment would be that a test revision project continues post-launch and only ends when the following revised edition is launched. This implies a continuous cycle of responsibility for a test from all its stakeholders that requires cooperation and collaboration in order for the test to succeed.

### Conclusion

The present study placed a spotlight on test revision to highlight its uniqueness from test development and adaptation, and the challenges faced by revision teams. By developing guidelines specific to test revisions and the use of revised psychological tests the study aimed to address calls over the last 20 years for such guidelines. The guidelines cover the lifespan of a test’s revision and will therefore be useful to revision teams, practitioners who participate in specific aspects of a test revision project, and the users of revised psychological tests.
